# The rs7044343 Polymorphism of the Interleukin 33 Gene Is Associated with Decreased Risk of Developing Premature Coronary Artery Disease and Central Obesity, and Could Be Involved in Regulating the Production of IL-33

**DOI:** 10.1371/journal.pone.0168828

**Published:** 2017-01-03

**Authors:** Javier Angeles-Martínez, Rosalinda Posadas-Sánchez, Luis Llorente, Edith Alvarez-León, Julian Ramírez-Bello, Teresa Villarreal-Molina, Guadalupe Lima, Guillermo Cardoso-Saldaña, José Manuel Rodríguez-Pérez, Nonanzit Pérez-Hernández, José Manuel Fragoso, Carlos Posadas-Romero, Gilberto Vargas-Alarcón

**Affiliations:** 1 Department of Molecular Biology, Instituto Nacional de Cardiología Ignacio Chávez, Mexico City, Mexico; 2 Department of Endocrinology, Instituto Nacional de Cardiología Ignacio Chávez, Mexico City, México; 3 Department of Immunology and Rheumatology, Instituto Nacional de Ciencias Médicas y Nutrición Salvador Zubirán, Mexico City, Mexico; 4 Histocompatibility Laboratory, Research Unit, Hospital Juárez de México, Mexico City, Mexico; 5 Cardiovascular Genomics Laboratory, Instituto Nacional de Medicina Genómica (INMEGEN), Mexico City, Mexico; China Medical University, TAIWAN

## Abstract

**Aim:**

The effect of interleukin 33 (IL-33) in the inflammatory process generates significant interest in the potential significance of IL-33 as a biomarker for coronary artery disease (CAD). Here, our objective was to analyze whether IL-33 gene polymorphisms are associated with premature CAD in a case-control association study.

**Methods:**

Four *IL-33* polymorphisms (rs7848215, rs16924144, rs16924159 and rs7044343) were genotyped by 5’ exonuclease TaqMan assays in 1095 patients with premature CAD and 1118 controls.

**Results:**

The rs7044343 *T* allele was significantly associated with a diminished risk of premature CAD (OR = 0.81, 95% CI: 0.69–0.97, P_dom_ = 0.020; OR = 0.85, 95% CI: 0.75–0.96, P_add_ = 0.019) and central obesity (OR = 0.74, 95% CI: 0.58–0.93, P_dom_ = 0.0007), respectively. When patients were divided into groups with and without type 2 diabetes mellitus (T2DM), the rs7044343 *T* allele was associated with a reduced risk of premature CAD in patients without (OR = 0.85, 95% CI: 0.73–0.99, P_add_ = 0.038) and with T2DM (OR = 0.61, 95% CI: 0.38–0.97, P_dom_ = 0.039; OR = 0.69, 95% CI: 0.49–0.97, P_add_ = 0.035). In order to establish the functional effect of the rs7044343 polymorphism, the production of IL-33 was determined in monocytes of selected individuals. Monocytes from individuals with rs7044343 *CC* genotype produced higher levels of IL-33 than monocytes from individuals with other genotypes.

**Conclusion:**

The results suggest that the *IL-33* rs7044343 *T* allele could be a susceptibility marker for premature CAD and central obesity. The rs7044343 polymorphism could be involved in regulating the production of IL-33.

## Introduction

Coronary artery disease (CAD) is a complex multifactorial disorder. This polygenic disease is caused by an inordinate inflammatory response to different forms of injuries to the arterial wall endothelium [1–3]. Admittedly, inflammation is as leading cause of atherogenesis since it disturbs lipoprotein metabolism and arterial wall biology. Infiltrates of T cells and activated macrophages are salient in atherosclerotic lesions of both humans and murines [4, 5]. The majority of T cells present in human atherosclerotic plaques belong to the CD4+ subset and produce predominantly cytokines of the Th1 subtype that have a critical pathogenic role in murine atherosclerosis models [6–10]. In contrast, it has been reported that the Th2 cells have an atheroprotective effect [11, 12].

IL-33 is a cytokine member of the IL-1 family, which includes IL-1 and IL-18 [13]. Unlike IL-1 and IL-18, which mainly promote Th1-associated responses, IL-33 predominantly induces the production of Th2 cytokines (IL-5 and IL-13) [14]. Miller et al. showed that IL-33 administration to ApoE-/- mice induced Th2 cytokines and protective ox-LDL antibodies, which significantly reduced atherosclerotic plaque development in the aortic sinus [15]. These data suggest that the gene that encodes IL-33 could be an important candidate gene for study in atherosclerosis. Recently, Tu et al. studied three IL-33 Tag SNPs (rs7025417, rs10975514, and rs10975519) in patients with CAD from the Chinese Han population [16]. In this study, the rs7025417 polymorphism was associated with CAD, with altered regulation of *IL-33* gene expression and with high plasma IL-33 levels. Results of association studies may vary between populations due to genetic differences amongst them, including differences in allele frequencies and linkage disequilibrium (LD) structures. Therefore, it is important to examine multiple ethnic populations for the identification of ethnicity-specific loci as well as common susceptibility loci. The objective of our study was to evaluate whether *IL-33* gene polymorphisms are associated with premature CAD in the Genetics of Atherosclerotic Disease (GEA) case-control association study. Also, the aim was to establish the possible effect of the associated polymorphism in the production of IL-33 in monocytes of individuals with different genotypes. After a functional prediction analysis, we selected four IL-33 gene polymorphisms (rs7848215, rs16924144, rs16924159, and rs7044343) with possible functional consequences and with minor allele frequency > 5% to be analyzed in the present study. The functional analysis showed that the rs7848215 produces a DNA binding site for the PBX1 transcription factor, the rs16924144 for SF/ASF, the rs16924159 for the SRp40 protein and the rs7044343 polymorphism produces binding site for the transcription factors SC35 and SF/ASF.

## Material and Methods

### Subjects

Every participant signed a written informed consent document. This protocol complies with the Declaration of Helsinki and was approved by the Ethics Committee of the Instituto Nacional de Cardiología Ignacio Chávez (INCICH). The GEA study focuses on the Mexican population and its main objective is to establish genetic factors linked with premature CAD and other coronary risk factors. All GEA study subjects are not blood related and are Mexican mestizos, who are defined as people born in Mexico, with an ancestry comprised of both indigenous inhabitants and individuals of African and/or Caucasian origin (mainly Spaniards), who had migrated to the Americas from the sixteenth century onward. A total of 2213 individuals were recruited, 1095 diagnosed with premature CAD and 1118 apparently healthy controls. History of myocardial infarction, angioplasty, revascularization surgery or coronary stenosis >50% on angiography (diagnosed before age 55 in men and before age 65 in women) was used to characterize premature CAD. Controls were seemingly healthy asymptomatic subjects without premature CAD family history, recruited from blood banks and Social Services centers. Congestive heart failure, liver, renal, thyroid or oncological disease were the exclusion criteria for controls. In an earlier report, we documented the selection of patients and controls of the GEA study [17]. Demographic, clinical, anthropometric, biochemical parameters and cardiovascular risk factors were assessed in all subjects. Qualified staff measured waist circumference, body mass index (BMI, kg/m^2^) and other anthropometric parameters. A sphygmomanometer was used to determine blood pressure (the average of the last two of three assessments). Patients with BMI ≥30 kg/m^2^ were classified as obese. Adult Treatment Panel III (ATP-III) criteria 2002 (Third report of the National Cholesterol Education Program) definitions were followed for central obesity, hypoalphalipoproteinemia, hypertriglyceridemia, and metabolic syndrome [18]. Total cholesterol (TC) levels ≥200 mg/dL defined hypercholesterolemia. Patients with systolic blood pressure ≥140 mmHg and/or diastolic blood pressure ≥90 mmHg, or the use of oral antihypertensive therapy were labeled as hypertense. And finally, we followed World Health Organization criteria to diagnose type 2 Diabetes mellitus (T2DM).

### Computed tomography of the chest and abdomen

Experienced radiologists interpreted the computed tomography of the chest and abdomen, performed using a 64-channel multi-detector helical computed tomography system (Somatom Sensation, Siemens). Coronary artery calcification (CAC) score was calculated using the Agatston method [19]. Total abdominal, subcutaneous and visceral adipose tissue areas (as described by Kvist et al.) were measured to assess the visceral to subcutaneous adipose tissue ratio (VAT/SAT) [20]. The hepatic to splenic attenuation ratio (LSAR) was estimated as described by Longo et al. [21]. CAC, VAT/SAT and LSAR were quantified using tomography scans. All patients and 1523 healthy controls underwent tomography. 405 controls were not considered for analysis, since their CAC score was positive, and they were thus considered as individuals with subclinical atherosclerosis (SA). The final control group included individuals (n = 1118) with only negative CAC scores.

### Genetic analysis

We isolated genomic DNA from whole blood containing EDTA using standard techniques. The rs7848215, rs16924144, rs16924159, and rs7044343 *IL-33* single nucleotide polymorphisms (SNPs) were genotyped using 5’ exonuclease TaqMan assays on an ABI Prism 7900HT Fast Real-Time PCR system (Applied Biosystems, Foster City, CA, USA). Genotyping call rate surpassed 95% for all SNPs tested, with no discordant genotypes in 10% of duplicate samples. We adhered to the manufacturer’s instructions to perform the assays.

Because the Mexican-Mestizo population is admixed, in order to assess the possible influence of population stratification, a panel of 265 ancestry informative markers (AIMs) distinguishing mainly Amerindian, European and African ancestry were selected [22] and genotyped on Illumina BeadStation using the GoldenGate assay. Duplicate control samples were genotyped on each chip, which also served as internal controls for quality of clustering and reproducibility. The primary analysis of the genotyping data with the Illumina Genome Studio software v.2011.1 was followed by visual inspection and assessment of data quality and clustering. Genotyping accuracy was also assessed by genotype clustering using the Illumina GeneTrain score, which is a measure of the clustering confidence of individual SNP alleles. Global Caucasian, Amerindian and African ancestry were determined in each individual using the ADMIXTURE software.

### Functional prediction analysis

The effect of the *IL-33* SNPs was predicted using the following bioinformatics software: FastSNP [23], SNP Function Prediction (http://snpinfo.niehs.nih.gov/snpfunc.htm), Human-transcriptome Database for Alternative Splicing (http://www.h-invitational.jp/h-dbas/), Splice Port: An Interactive Splice Site Analysis Tool (http://www.spliceport.cs.umd.edu/SplicingAnalyser2.html), ESE finder (http://rulai.cshl.edu/cgi-bin/tools/ESE3/esefinder.cgi), HSF (http://www.umd.be/HSF/), and SNPs3D (http://www.snps3d.org/).

### Monocyte isolation

A sample of venous blood (40 mL) was obtained from 61 healthy controls selected according to the rs7044343 polymorphism (21 with *CC*, 21 with *TC* and 21 with *TT* genotypes). The peripheral blood mononuclear cell (PBMC) population was isolated by gradient centrifugation on Lymphoprep (Axis-Shield PoC AS, Oslo, Norway). Monocytes were isolated by positive selection with CD14-mAb-coated micro beads (Miltenyi Biotec, Bergisch Gladbach; Germany) following the manufacturer’s instructions (purity of 95–98%).

### Monocyte cultures and IL-33 detection

Monocytes were counted in a Neubauer hemocytometer (Propper MFG Company, NY USA) chamber using 0.4% Trypan blue stain (Cambrex Bio Science, MD USA) to exclude dead cells. Monocyte density in culture was adjusted to 1 x10^6^ per milliliter. Monocytes were cultured in RPMI-1640 medium (Sigma, Poole, UK), supplemented with 10% (volume/volume) heat-inactivated fetal bovine serum (Sigma), 0.1 mM L-glutamine, 100 U/ml penicillin and 100 U/ml streptomycin. Cells were stimulated with 100 ng/ml *E coli* lipopolysaccharide (from strain 0111:B4, Sigma) and 100 ng/ml of *P gingivalis* lipopolysaccharide (Invivogen, Calne, UK) for 6 hours in a humidified atmosphere with 5% CO2 at 37°C. Necrosis was induced by subjecting stimulated cells to five cycles of freezing to -70°C and thawing at 38°C. Necrotic cell preparations were centrifuged at 10,000 for 5 min and supernatants were kept at -70°C. The IL-33 levels were detected using specific ELISA kit for IL-33 (Biolegend, San Diego, CA); the sensitivity for the ELISA was 4.14 pg/ml.

### Statistical analysis

The SPSS version 18.0 statistical package (SPSS, Chicago, Il) was employed for the statistical estimation of means ± SD and frequencies of baseline characteristics. We compared frequencies using Chi-square tests, and means using the ANOVA and Students t-test. To determine the association between the polymorphisms and metabolic variables, we used ANCOVA and adjusted for age, gender, BMI, smoking history and alcohol consumption. The correlation of polymorphisms with premature CAD under dominant, recessive and additive inheritance models was analyzed with logistic regression analysis. Also, we utilized age, gender, BMI, smoking history, alcohol consumption and ancestry to adapt the models. They were constructed including one variable at time, and final models included variables with biological relevance or with statistical significance or both. Confounding bias was accepted when changes in estimated odds ratios (ORs) were equal or greater than 10%. When a principal effect model was reached, effect modification was also tested and interactions terms were constructed between the polymorphisms and different variables; the terms were included in the model when the significance of the p-value was greater or equal to 0.20. Hosmer–Lemeshow Goodness of Fit test was performed for each multiple logistic model. Bonferroni correction was used as appropriate. Statistical power to detect association with CAD was 0.80 as estimated with QUANTO software [http://hydra.usc.edu/GxE/]. The obtained genotype frequencies did not deviate from Hardy-Weinberg equilibrium (HWE, P > 0.05). Haploview version 4:1 (Broad Institute of Massachusetts Institute of Technology and Harvard University, Cambridge, MA, USA) was used to calculate pairwise linkage disequilibrium (LD, D´) between polymorphisms and haplotype reconstruction.

## Results

Tables 1 and 2 illustrate the general characteristics of the study population. Global ancestry was similar in patients and controls, showing 55.8% and 54.0% of Native American ancestry respectively; and 34.3% and 35.8% of Caucasian ancestry, respectively.

**Table 1:** Demographic characteristics of the population.

		Premature CAD (n = 1095)			Controls (n = 1118)			P value
		P25	Median	P75	P25	Median	P75	
Age (years)		49	54	59	45.0	51.0	57.0	<0.0001
Body Mass Index (Kg/m^2^)		26.0	28.3	31.3	25.4	27.9	31.0	0.006
Waist circumference (cm)		91.2	97.5	105.4	86.0	94.0	101.5	<0.0001
Total Abdominal Fat (cm2)		340	426	530.25	346	443	545	0.186
Subcutaneous Abdominal Fat (cm2)		193	245.5	316	219.3	288.5	373.0	<0.0001
Visceral Abdominal Fat (cm2)		130	171	218.5	104	142	183	<0.0001
Visceral/Subcutaneous adipose tissue ratio		0.95	1.29	1.81	0.53	1.26	2.29	0.031
Blood Pressure (mmHg)	Systolic	107	116.3	128	105.3	114.6	125.0	0.002
	Diastolic	66	71.7	78.5	66.0	71.3	77.3	0.235
Heart Rate (bpm)		57.5	64.3	72.7	59.5	65.3	71.0	0.158
Gender n (%)	Male	905 (82.6)			456 (40.8)			
	Female	192 (17.4)			662 (59.2)			<0.0001
Weight n (%)	Normal weight	190 (17.4)			259 (23.2)			
	Overweight	515 (47)			508 (45.4)			0.005
	Obesity	390 (35.6)			351 (31.4)			<0.000
Central obesity n (%)		866 (79.2)			868 (77.7)			0.263
Tobacco smoking n (%)	Current	135 (12.3)			250 (22.4)			<0.0001
	Former	702 (65.4)			346 (35.2)			<0.0001
Use of alcohol n (%)		610 (55.7)			823 (73.8)			<0.0001
Hypertension n (%)		740 (67.6)			302 (27.0)			<0.0001
Hypertensive Medication n (%)		737 (71.3)			170 (15.2)			<0.0001

Data are expressed as median and percentiles 25 and 75.

*P values were estimated using Mann-Whitney U-test continuous variables and Chi-square or Fisher test for categorical values.

**Table 2:** Comparison of biochemical parameters in individuals with premature CAD and healthy controls.

	CAD premature			Controls			P value
	P25	Median	P75	P25	Median	P75	
Total cholesterol (mg/dl)	132.50	160.70	193.60	168.08	190.05	210.00	<0.0001
HDL-C (mg/dl)	32.50	38.30	45.05	36.93	46.00	56.00	<0.0001
LDL-C (mg/dl)	68.70	91.00	116.00	96.16	115.76	133.67	<0.0001
Triglycerides (mg/dl)	119.00	162.80	221.60	108.10	143.20	199.50	<0.0001
ApoA1 (mg/dl)	63	79	102	73	90	108	<0.0001
ApoB (mg/dl)	102.00	120.00	136.90	114	134	157	<0.0001
Glucose (mg/dl)	87	95	120	84	90	97	<0.0001
Insulin	14.84	20.04	28.19	12.59	17.51	24.04	<0.0001
HOMA	3.53	5.15	7.86	2.70	3.94	5.68	<0.0001
Alanine transaminase (IU/L)	19.0	26.0	36.0	17.0	23.0	33.0	0.002
Aspartate transaminase (IU/L)	22.0	26.0	31.0	21.0	25.0	30.0	0.002
Alkaline Phosphatase (IU/L)	64.0	77.0	95.0	68.0	81.0	97.8	0.001
Gamma-glutamyl transpeptidase (IU/L)	23.0	33.0	50.0	17.0	25.0	41.0	<0.0001
TC > 200 mg/dL n (%)	228 (20.8)			401 (35.9)			<0.0001
Hypo-a-lipoproteinemia n (%)	696 (63.6)			565 (50.6)			<0.0001
Hypertriglyceridemia n (%)	626 (57.2)			520 (46.6)			<0.0001
Type 2 Diabetes Mellitus n (%)	414 (37.8)			109 (9.7)			<0.0001
Metabolic Syndrome n (%)	551 (50.3)			450 (40.3)			<0.0001

Data are expressed as median and percentiles 25 and 75.

*P values were estimated using Mann-Whitney U-test continuous variables and Chi-square or Fisher test for categorical values.

### Association of polymorphisms with premature CAD

Genotype frequencies in the polymorphic sites were in HWE. In all the evaluated models, the distribution of rs16924144, rs16924159, and rs7848215 polymorphisms was comparable in premature CAD patients and healthy controls. Conversely, the distribution of rs7044343 was not the same in the investigated groups. The rs7044343 *T* allele was associated with diminished risk of premature CAD when contrasted with to healthy controls (OR = 0.81, 95% CI = 0.69–0.97, P_dom_ = 0.020; OR = 0.85, 95% CI: 0.75–0.96, P_add_ = 0.019)) under dominant and additive models adjusted for age, gender, BMI, smoking history, alcohol consumption and ancestry (Table 3).

**Table 3:** Associations of *IL33* polymorphisms with premature CAD.

Polymorphism	Alleles	MAF^a^	MAF^a^	Genotypes	Genotypes	P_hwe_	OR (95% CI)
		CAD	Control	Premature CAD	Control	CAD/Control	P_dom_ value
rs16924144	*C**/T*	0.48	0.48	281/564/250	294/569/255	0.33/0.55	0.85 (0.62–1.18); 0.34
				0.25/0.52/0.23	0.26/0.51/0.23		1.00 (0.73–1.38); 0.97^b^ 0.94 (0.77–1.15); 0.57^c^
rs16924159	*A**/G*	0.49	0.49	226/448/224	238/471/227	0.95/0.90	1.07 (0.74–1.55); 0.69
				0.25/0.50/0.25	0.25/0.50/0.24		1.06 (0.72–1.56); 0.75^b^ 1.05 (0.83–1.32); 0.66^c^
rs7848215	*C/**T*	0.14	0.14	827/238/30	832/263/23	0.01/0.71	0.91 (0.67–1.24); 0.58
				0.75/0.22/0.03	0.74/0.24/0.02		1.60 (0.66–3.84); 0.29^b^ 0.98 (0.75–1.27); 0.88^c^
rs7044343	*C/**T*	0.33	0.37	481/492/122	437/540/141	0.84/0.22	**0.81 (0.69–0.97); 0.020**
				0.44/0.45/0.11	0.39/0.48/0.13		0.84 (0.56–1.27); 0.420 ^b^
							**0.85 (0.75–0.96); 0.019** ^c^

Adjusted for age, gender, BMI, smoking history, alcohol consumption and ancestry.

^a^: MAF, minor allele frequency.

^b^: recessive model.

^c^: additive model.

CAD premature: Coronary artery disease premature.

Phwe: p value from Hardy-Weinberg equilibrium tests.

NS: Not significant.

*Underlined letter denotes the minor allele in the control samples.

Significant values are in bold.

The p values were corrected multiplying by 4, number of SNPs tested.

### Association of the polymorphisms with cardiovascular risk factors

We considered the association of rs16924144, rs16924159, rs7848215 and rs7044343 polymorphisms with cardiovascular risk factors by comparing CAD patients and healthy controls. Under dominant model adjusted by age, gender, BMI, smoking history and alcohol consumption, the rs7044343 polymorphism was associated with reduced risk of central obesity (OR = 0.74, 95% CI = 0.58–0.93, P_dom_ = 0.0007) (Table 4).

**Table 4:** Association of the rs7044343 polymorphism with metabolic risk factors.

	Dominant model	Premature CAD	Control	OR (95% CI)	P value
Obesity *n* (%)	*C/C*	162 (0.15)	124 (0.11)		
	*C/T+TT*	221 (0.20)	179 (0.16)	NS	-
Central obesity	*C/C*	372 (0.34)	286 (0.26)		
	*C/T+TT*	470 (0.43)	475 (0.42)	**0.74 (0.58–0.93)**	**0.0007**
Hypo-α-lipoproteinemia *n* (%)	*C/C*	287 (0.26)	199 (0.18)		
	*C/T+TT*	386 (0.35)	287 (0.26)	NS	-
Hypercholesterolemia *n* (%)	*C/C*	102 (0.09)	120 (0.11)		
	*C/T+TT*	121 (0.11)	225 (0.20)	NS	-
Hypertriglyceridemia *n* (%)	*C/C*	264 (0.24)	173 (0.15)		
	*C/T+TT*	346 (0.32)	271 (0.24)	NS	-
Metabolic syndrome *n* (%)	*C/C*	229 (0.21)	163 (0.15)		
	*C/T+TT*	307 (0.28)	228 (0.20)	NS	-
Type 2 diabetes mellitus *n* (%)	*C/C*	176 (0.16)	51 (0.05)		
	*C/T+TT*	226 (0.21)	49 (0.04)	NS	-

The model was adjusted by age, gender, BMI, smoking history and alcohol consumption.

NS: Not significant.

OR: Odds ratio.

CI: Confidence intervals.

Significant values are in bold.

The dominant model was analyzed considering 7 metabolic risk factors, so the p values were corrected multiplying by 7.

### Association of the polymorphisms with metabolic parameters

We evaluated the effect of rs16924144, rs16924159, rs7848215 and rs7044343 polymorphisms on numerous metabolic parameters separately in controls (CAC score = 0), and premature CAD subjects. None of the studied polymorphisms was associated with metabolic parameters in the groups.

### Association of the polymorphisms with premature CAD in patients with and without diabetes mellitus

Considering the high frequency of diabetes mellitus in our group of patients with CAD, we carried out an analysis in patients with and without this pathology in order to establish if the polymorphisms are associated with CAD or with T2DM. The rs7044343 *T* allele was associated with decreased risk of CAD in patients without T2DM (OR = 0.85, 95% CI = 0.73–0.99, P_add_ = 0.038) (Table 5) and with T2DM (OR = 0.61, 95% CI = 0.38–0.97, P_dom_ = 0.039; OR = 0.69, 95% CI = 0.49–0.97, P_add_ = 0.035) (Table 6). The models were adjusted for age, gender, BMI, smoking history, alcohol consumption and ancestry.

**Table 5:** Associations of *IL33* polymorphisms in patients with premature CAD without diabetes mellitus.

Polymorphism	Alleles	MAF^a^	MAF^a^	Genotypes	Genotypes	P_hwe_	OR (95% CI)
		CAD	Control	Premature CAD	Control	CAD/Control	P_dom_ value
rs16924144	*C/**T*	0.49	0.48	168/355/159	240/460/211	0.45/0.38	0.86 (0.60–1.25); 0.45
				0.24/0.52/0.23	0.26/0.51/0.23		0.99 (0.69–1.42); 0.96^b^ 0.94 (0.75–1.18); 0.62^c^
rs16924159	*A**/G*	0.50	0.50	142/296/145	192/400/192	0.87/0.50	1.17 (0.77–1.78); 0.44
				0.24/0.51/0.25	0.25/0.51/0.24		1.06 (0.68–1.65); 0.77^b^ 1.09 (0.83–1.42); 0.51^c^
rs7848215	*C/**T*	0.15	0.14	500/162/20	678/213/20	0.09/0.52	0.97 (0.68–1.37); 0.88
				0.73/0.24/0.03	0.74/0.23/0.02		1.30 (0.51–3.35); 0.57^b^1.01 (0.74–1.35); 0.95^c^
rs7044343	*C/**T*	0.34	0.38	300/306/76	343/447/121	0.87/0.29	1.03 (0.74–1.45); 0.826
				0.44/0.45/0.11	0.38/0.49/0.13		0.81 (0.50–1.32); 0.404^b^
							**0.85 (0.73–0.99); 0.038**^c^

Adjusted for age, gender, BMI, smoking history, alcohol consumption and ancestry.

^a^: MAF, minor allele frequency.

^b^: recessive model.

^c^: additive model.

CAD premature: Coronary artery disease premature.

Phwe: p value from Hardy-Weinberg equilibrium tests.

NS: Not significant.

*Underlined letter denotes the minor allele in the control samples.

Significant values are in bold.

The p values were corrected multiplying by 4, number of SNPs tested.

**Table 6:** Associations of *IL33* polymorphisms in patients with premature CAD and diabetes mellitus.

Polymorphism	Alleles	MAF^a^	MAF^a^	Genotypes	Genotypes	P_hwe_	OR (95% CI)
		CAD	Control	Premature CAD	Control	CAD/Control	P_dom_ value
rs16924144	*C/**T*	0.47	0.49	109/207/86	167/358/162	0.55/0.13	0.77 (0.46–1.31); 0.34
				0.27/0.52/0.21	0.24/0.52/0.24		0.95 (0.56–1.61); 0.84^b^0.89 (0.64–1.23); 0.48^c^
rs16924159	*A**/G*	0.50	0.48	83/156/85	148/315/130	0.51/0.18	1.14 (0.64–2.04); 0.65
				0.26/0.48/0.26	0.25/0.53/0.22		1.31 (0.71–2.42); 0.39^b^ 1.16 (0.80–1.67); 0.42^c^
rs7848215	*C/**T*	0.12	0.15	316/75/11	500/172/15	0.03/1.00	0.68 (0.41–1.13);0.145
				0.79/0.19/0.03	0.73/0.25/0.02		0.96 (0.24–3.72); 0.95^b^
							0.75 (0.49–1.16); 0.202^c^
rs7044343	*C/**T*	0.33	0.40	176/185/41	235/351/101	0.66/0.26	**0.61 (0.38–0.97); 0.039**
				0.44/0.46/0.10	0.34/0.51/0.15		0.65 (0.34–1.27); 0.216^b^
							**0.69(0.49–0.97); 0.035**^c^

Adjusted for age, gender, BMI, smoking history, alcohol consumption and ancestry.

^a^: MAF, minor allele frequency.

^b^: recessive model.

^c^: additive model.

CAD premature: Coronary artery disease premature.

Phwe: p value from Hardy-Weinberg equilibrium tests.

NS: Not significant.

*Underlined letter denotes the minor allele in the control samples.

In this analysis the control group only included individuals without diabetes.

Significant values are in bold.

The p values were corrected multiplying by 4, number of SNPs tested.

### Haplotype analysis and SNP functional prediction

Even though the *IL-33* polymorphisms were in high linkage disequilibrium (D’>0.8 and r^2^>0.9), the distribution of the haplotypes in premature CAD patients and healthy controls was comparable (data not included).

Interestingly, SNP functional prediction software results suggest that the rs7044343 polymorphism is functional. The variation in the rs7044343 polymorphism produces a DNA binding site for the transcription factors SC35 and SF/ASF with possible consequences in the expression of the *IL-33*.

### IL-33 levels in monocytes

**Fig 1:**
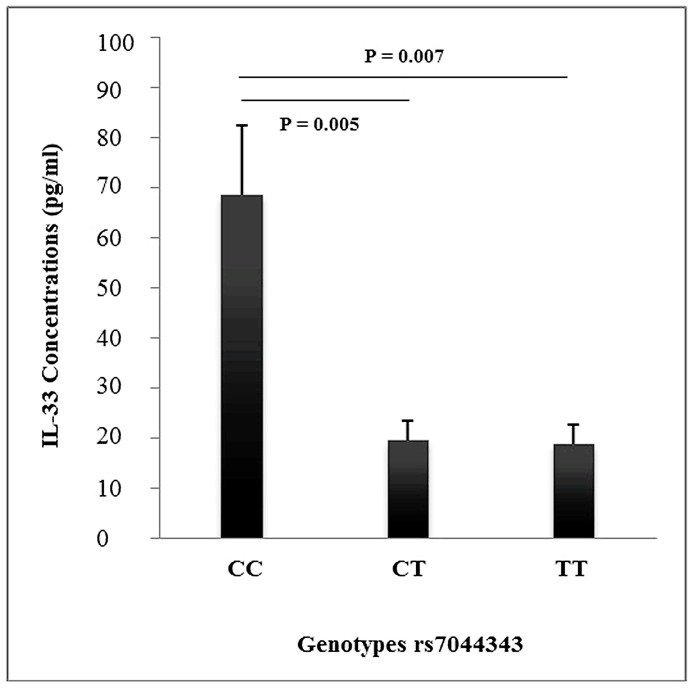
Detection of IL-33 in monocytes from healthy individuals with rs7044343 *CC*, *TC* and *TT* genotypes (21 participants were included for each genotype). Data are presented as mean ± SD. *CC* vs *CT* (P = 0.005) and *CC* vs *TT* (P = 0.007).

In order to establish the functional effect of the rs7044343 polymorphism, the production of IL-33 was determined in monocytes of selected individuals (Fig 1). Monocytes from individuals with rs7044343 *CC* genotype produced higher levels of IL-33 (32.08+24.30) than those from patients with *CT* (16.32 ± 6.23) (P = 0.005) and *TT* (17.10 ± 5.48) (P = 0.007) genotypes.

## Discussion

IL-33 is a cytokine with an important role in the inflammatory process and in the pathogenesis of atherosclerosis [15]. Animal studies have indicated that IL-33 reduces macrophage foam cell formation [24] and inhibits the development of atherosclerosis in apolipoprotein E-deficient mice [15]. In spite of the important role of IL-33 in the development of atherosclerosis, too few studies have explored the possible role of the gene that encodes this cytokine in the genetic susceptibility to coronary artery disease. Tu et al. reported the association of the *IL-33* rs7025417 polymorphism with the risk of developing CAD in a Chinese Han population, thus demonstrating an effect of this polymorphism in the *IL-33* gene expression and plasma levels [16]. In another work, four *IL-33* polymorphisms (rs1929992, rs10975520, rs11792633 and rs16924159) were studied in Chinese patients with CAD and none of them was associated with the disease [25]. Due to the effect of IL-33 in the inflammatory process, other polymorphisms in this gene have been associated with asthma, inflammatory bowel disease and Alzheimer’s disease [26–28]. In our study, four *IL-33* gene polymorphisms (rs7848215, rs16924144, rs16924159, and rs7044343) were analyzed in order to establish their role as a susceptibility marker for premature CAD. We selected these polymorphisms considering their possible functional effect after an informatics analysis and considering that they have a minor allele frequency major to 5%. In our study, the rs7044343 was associated with reduced risk of developing CAD in patients with and without diabetes mellitus. This polymorphism was also associated with reduced risk of developing central obesity. The association of the rs7044343 genotypes in some diseases is contradictory. In the study of Li et al., the rs7044343 *CC* genotype was associated with decreased risk of developing rheumatoid arthritis (RA) and with low serum IL-33 levels [29]. Contrary to that detected in RA, in systemic sclerosis, the rs7044343 *CC* genotype was associated with increased risk of developing this disease [30]. Our result of rs7044343 polymorphism contrasts with that reported by Li et al., [29] in RA, however, are in line with that reported by Koca et al., [30] in systemic sclerosis, because in our study, the rs7044343 *T* allele was significantly associated with a diminished risk of premature CAD. Also, in the study of Li et al., [29] the rs7044343 *CC* genotype was associated with low serum IL-33 levels in AR patients. This result contrasts with our report, because, we detected a higher production of IL-33 in monocytes of individuals with the *CC* genotype, compared to those carrying CT and TT genotypes. Some methodological differences between the two studies could explain the apparent contradictory results. In the study by Li et al., [29] the measures were made in serum of RA patients, whereas in our study, the measures were made in monocytes cultures of healthy controls. The functional prediction software used here predicted that this polymorphism is functional. The presence of the *C* allele in this polymorphism produces a binding site for the transcription factors SC35 and SF/ASF proteins. These proteins belong to the family of SR proteins that regulate alternative splicing [31]. This polymorphism could have functional effects increasing the production of IL-33 isoforms with the consequent increase of the anti-atherogenic effect of this cytokine. In order to establish the functional effect of the rs7044343 polymorphism, the production of IL-33 was determined in monocytes of selected individuals. Monocytes from individuals with rs7044343 *CC* genotype produced higher levels of IL-33 than monocytes from individuals with other genotypes, suggesting a role of this polymorphism in the production of IL-33. It should be mentioned that monocytes used for this analysis were obtained from healthy individuals (without CAD or CAC). Therefore, the fact that individuals with the *CC* genotype produce more IL-33 does not necessarily mean the development of CAD. Alternatively, the inflammatory process in CAD includes the participation of several both pro- and anti-inflammatory cytokines. This is a complex phenomenon in which IL-33 may be playing a very important role. The functional analysis on monocytes only was made considering the rs7044343 polymorphism because the main objective of the study was to analyze whether IL-33 gene polymorphisms are associated with premature CAD. The analysis in the whole group of CAD patients and in the group of CAD patients with and without T2DM confirms the association of the rs7044343 polymorphism with CAD.

As for the limitations herein, we only included the study of four polymorphisms of *IL-33*, which seem to be functional based on the analysis of the prediction software used. In our study, we do not analyze the expression and neither plasma levels of IL-33 in CAD patients and healthy controls. However, we consider that the evaluation of IL-33 production in monocyte cultures of individuals with different genotypes could be a more direct approach of the effect of these genotypes in the production of IL-33. Since this is the first work that documents the correlation of the *IL-33* polymorphisms with premature CAD and central obesity, further studies in an independent group of patients are required to validate the results. Indeed, a strength of our work is that the control group only included individuals without subclinical atherosclerosis (individuals without coronary artery calcification).

The *IL-33* polymorphisms were in strong linkage disequilibrium in the present work; and still, the haplotypes were not associated with premature CAD. Crawford et al. described that the haplotype architecture of candidate genes across the human genome is convoluted. Also, they mentioned that a considerable the amount of sequence variation has not been documented yet [32]. Consequently, the absence of association of *IL-33* haplotypes in our study is not definitive, owing to the incomplete knowledge of both the genetic variation within the *IL-33* gene and the structure of linkage disequilibrium in the analyzed region.

## Conclusions

In conclusion, the association of the *IL-33* rs7044343 polymorphism with both premature CAD and central obesity is established here. This polymorphism had functional effects, based on an *in silico* prediction analysis. In this study, we demonstrate that the rs7044343 polymorphism has an effect in the production of IL-33 in monocytes stimulated by lipopolysaccharide. Notably, Mexican people form a population with a distinctive genetic background and important differences [33–36]. Thus, owing to these genetic characteristics, the associations of the *IL-33* polymorphisms shown here are not definitive and should be tested in other independent populations.
